# Glasgow prognostic score is an independent marker for poor prognosis with all cases of epithelial ovarian cancer

**DOI:** 10.1002/cam4.681

**Published:** 2016-03-01

**Authors:** Chiaki Omichi, Keiichiro Nakamura, Junko Haraga, Hisashi Masuyama, Yuji Hiramatsu

**Affiliations:** ^1^ Department of Obstetrics and GynecologyOkayama University Graduate School of Medicine, Dentistry and Pharmaceutical SciencesOkayamaJapan

**Keywords:** Glasgow prognostic score, ovarian cancer, predictor for poor

## Abstract

Inflammatory markers are important prognostic factors in various cancers. This study investigated whether inflammatory markers of the Glasgow prognostic score (GPS) predicted progression‐free survival (PFS) and overall survival (OS) for patients with all cases of epithelial ovarian cancer (OC). Pretreatment GPS was examined for the correlations with PFS and OS in 216 patients in all stages of epithelial OC. Statistical analyses were performed using the Mann–Whitney *U*‐test. PFS and OS were analyzed using the Kaplan–Meier method. Cox's proportional hazard regression was used for univariate and multivariate analyses. For all patients, the median PFS was 35.1 months, and median OS was 46.7 months; follow‐up range was 1–162 months. Kaplan–Meier analysis revealed that patients with high GPS (GPS 2) at pretreatment had a shorter PFS and OS than did patients with lower GPS (GPS 0 + 1) in for early, advanced, and all‐stages of OC (PFS:* P* < 0.001 for early‐, advanced‐ and all‐stages; OS;* P *< 0.001 for early‐ and all‐stage, *P* = 0.015 for advanced‐stage). GPS (GPS 2) was also found to be an independent predictor of both recurrence (*P* = 0.002) and survival (*P* = 0.001) of all cases of epithelial OC by a multivariate analysis. GPS can serve as an indicator of poor prognosis in patients with all stages of epithelial OC, including early‐stage disease and regardless of histology.

## Introduction

Ovarian cancer (OC) is the second most common gynecological malignancy in the United States; it accounted for about 21,500 new cases of cancer and 14,600 deaths in the United States in 2009 1. In Japan, 8000 cases of OC are newly diagnosed and more than 4000 women die of the disease every year 2. Its 5‐year survival rate is inversely related to the disease stage at first diagnosis. Although the 5‐year survival rate for stage I disease is 92.7%, most cases (67–74%) are diagnosed with metastatic disease (stage III–IV), which has a 5‐year survival rate of only 30.6% 3. Known prognostic factors for OC include residual tumor and chemotherapy response 4, 5, but these parameters are not sufficient to predict accurate OC prognoses. Therefore, a new approach for pretreatment assessment of OC is pivotal in improving outcomes.

Inflammatory markers are important prognostic factors for survival in various cancer types. C‐reactive protein (CRP) and albumin play prominent roles in tumor inflammation 6, 7, 8. Reportedly, inflammation‐based prognostic scores, including the Glasgow prognostic score (GPS)—a combination of CRP and albumin levels—is associated with survival in various cancers, including lung, breast, esophagus, stomach, pancreas, kidney, and colorectal cancers 9, 10, 11, 12, 13, 14, 15. Although data on survival outcomes has in advanced OC has been published 16, patients with early‐stage OCs have not been sufficiently investigated. In this study, we investigated the correlation between pretreatment GPS and prognosis of patients with all stages of OC including those with early‐stage epithelial OC.

## Methods

### Study population

This retrospective study reviewed medical records of 216 patients with different stages (stages I–IV) of epithelial OC who were treated at the Department of Obstetrics and Gynecology of Okayama University Hospital between January 2002 and July 2015. The study protocol was approved by the Institutional Review Board of Okayama University Hospital. All patients gave informed consent. Staging of disease was done according to the FIGO criteria for ovarian carcinoma. All 216 patients had to have a diagnosis of stage on the basis of imaging or surgical finding. The use of computed tomography (CT)/positron emission tomography/CT (PET‐CT) to locate tumor deposits before debulking surgery has become standard practice. An attempt is made to identify signs of transdiaphragmatic tumor spread, such as diffuse peritoneal thickening, large‐volume ascites, large bowel involvement, diaphragmatic disease, splenic involvement, hepatic involvement, bulky omental disease, pleural space/extra‐abdominal disease, on chest, and abdominal CT/PET‐CT images because their presence may have a substantial effect on further management. Lymph node with short‐axis lengths >10.0 mm were defined as metastatic with CT/PET‐CT in the interval debulking surgery (IDS) group. The amount of ascites >500 mL was defined as present in the both Primary debulking surgery (PDS) and IDS group 17, 18.

PDS was performed if in the opinion of the multidisciplinary team, consisting of gynecologic oncologists, medical oncologists, and a dedicated radiologist, debulking surgery of all visible tumor to less than one centimeter in diameter was possible. Every operative cytoreductive procedure was performed with the aim of leaving complete resection with no residual tumor (R0). All PDS cases were successfully performed R0. Patients with more extensive disease and those unable to undergo surgery started neoadjuvant chemotherapy. Patients who underwent exploratory laparotomy for diagnostic biopsy or oophorectomy without debulking were analysed in the IDS group. Surgical resection was classified as curative (R0, complete resection with no residual tumor) or noncurative (R1 or R2, microscopic or gross residual tumor) on IDS group. Patients who underwent PDS were then treated with or without 3–6 cycles of standard chemotherapy (*n* = 115), including 3–6 cycles of neoadjuvant chemotherapy with IDS followed by 2–3 cycles of adjuvant chemotherapy (*n* = 91). All (early‐and advanced‐stage) of the patients underwent a laparotomy for total abdominal hysterectomy, bilateral salpingo‐oophorectomy and omentectomy with or without pelvic and/or para‐aortic lymphadenectomy. Pelvic lymph node (PLN) dissection included the right and left common iliac, external iliac, suprainguinal, internal iliac, obturator, sacral, and parametrial nodal chains. Para‐aortic lymph node (PAN) dissection included the nodes located from the bifurcation of the aorta to the level of the renal vein.

### Laboratory data collection

All subjects had serum albumin, CRP, and CA125 levels recorded within 1 week before their treatments. Levels of serum albumin and CRP were measured using latex nephelometry (LT Auto Wako, Osaka, Japan). Serum CA125 level was measured with electrochemiluminescence immunoassay on the Roche/Hitachi Modular Analysis E170 (Roche Diagnostics, Tokyo, Japan). GPS was estimated as described previously 9. Briefly, the high GPS group included patients with GPS 2: both CRP levels >1.0 mg/dL and hypoalbuminemia (<3.5 g/dL). The low GPS group included patients with only one of these abnormal levels (GPS 1) or none of these abnormalities (GPS 0).

### Statistical analysis

Statistical analyses were performed using the Mann–Whitney *U*‐test for comparisons with controls. Progression‐free survival (PFS) and overall survival (OS) of the groups were analyzed using the Kaplan–Meier method. Differences between the recurrence and survival curves were examined using the log‐rank test. We performed univariate and multivariate analyses using Cox's proportional hazards model to determine which factors predict PFS and OS after adjusting for effects of known prognostic factors. Analyses were performed using SPSS software version 20.0 (SPSS Inc., Chicago, IL). *P *< 0.05 was considered statistically significant.

## Results

The patients were aged 16–81 years (median: 61.0 years); their median pretreatment CRP: 3.5 mg/dL (range: 0–54.34 mg/dL); albumin: 3.22 g/dL (2.3–4.8 g/dL); and CA125: 307.6 U/mL (6.4–22594 U/mL). Patients’ ages, FIGO stage, histology, lymph node metastasis, lymphadenectomy, no residual tumor (R0) and neoadjuvant chemotherapy are shown in Table 1.

**Table 1:** Patient and tumor characteristics

Baseline characteristics	All patients	
Age at diagnosis, y	Mean, 61.0; range, 16–81	
	Numbers	*N* (%)
Stage		
I	87	40.3
II	15	6.9
III	88	40.7
IV	26	12.1
Histology		
Serous adenocarcinoma	113	52.3
Clear cell carcinoma	31	14.4
Mucinous adenocarcinoma	25	11.6
Endometrioid adenocarcinoma	26	12
Other carcinoma	5	2.3
Mixed type carcinoma	16	7.4
Lymph node metastasis		
Absent	144	66.7
Present	72	33.3
Lymphadenectomy		
Absent	130	60.2
Present	86	39.8
Macroscopic tumor free (R0)		
Absent	17	7.9
Ascites		
Absent	181	83.8
Present	35	16.2
Neoadjuvant chemotherapy		
Absent	125	57.9
Present	91	42.1

We examined pretreatment GPS in patients with early‐ and/or advanced‐stage epithelial OC. Their pretreatment GPS were GPS 0: 127 patients (58.8%); GPS 1: 55 (25.4%); GPS 2: 34 (15.8%). The cut‐off value for the CA125 level determined from the median values was 307.6 U/mL, respectively. We also found pretreatment GPS was significantly associated with stage (*P *< 0.001), histology (*P *= 0.001), lymph node metastasis (*P *< 0.001), lymphadenectomy (*P *< 0.001), no residual tumor (R0) (*P *= 0.006), ascites (*P *< 0.001), neoadjuvant chemotherapy (*P *< 0.001), and CA125 (*P *< 0.001) (Mann–Whitney *U*‐test, *P *< 0.05; Table 2).

**Table 2:** Associations of GPS with clinical factors on ovarian cancer

Variable	Numbers of GPS 0	Numbers of GPS 1	Numbers of GPS 2	*P*‐value
Stage				<0.001^a^
I–II	77	20	5	
III–IV	50	35	29	
Histology				0.001^a^
Serous adenocarcinoma	55	31	27	
Non‐Serous adenocarcinoma	72	24	7	
Lymph node metastasis				<0.001^a^
Absent	103	27	14	
Present	24	28	20	
Lymphadenectomy				<0.001^a^
Absent	85	27	18	
Present	42	28	16	
No residual tumor (R0)				0.006^a^
Absent	4	7	6	
Present	123	48	28	
Ascites				<0.001^a^
Absent	123	40	18	
Present	4	15	16	
Neoadjuvant chemotherapy				<0.001^a^
Absent	95	22	8	
Present	32	33	26	
CA125				<0.001^a^
≤307.6 U/mL	85	19	4	
>307.6 U/mL	42	36	30	

GPS, glasgow prognostic score.

a *P* < 0.05.

**Figure 1:**
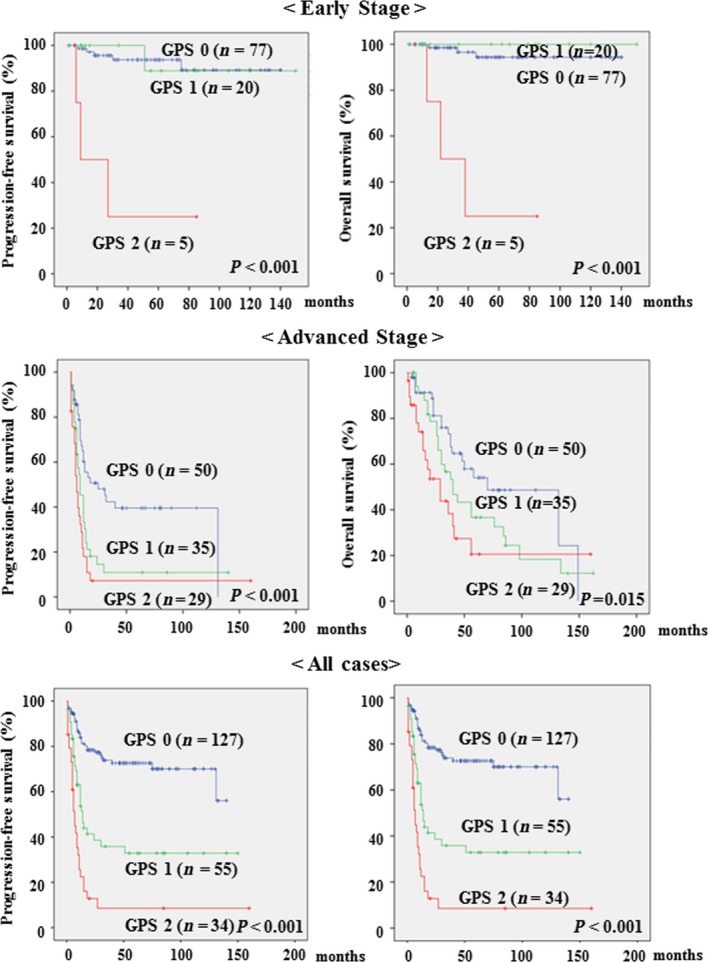
Kaplan–Meier curves for progression‐free survival and overall survival (OS) rates of 216 patients with ovarian cancer (OC) according to their Glasgow prognostic score (GPS) at pretreatment. Early stage (Stages I–II): GPS score 0 (*n *= 77); GPS 1 (*n *= 20); GPS 2 (*n *= 5): Advanced stage (Stages III–IV): GPS score 0 (*n *= 50); GPS 1 (*n *= 35); GPS 2 (*n *= 29): All cases of OC (Stages I–IV); GPS score 0 (*n *= 127); GPS 1 (*n *= 55); GPS 2 (*n *= 34).

Patients had follow‐up examinations approximately every 1–2 months for first 6 months, every 3 months for next 2 years, and every 6 months thereafter. For all patients, median PFS was 35.1 months OS was 46.7 months; follow‐up range was 1–162 months (for both OS and PFS). At the time of last follow‐up, 124 patients were alive with no evidence of disease, 69 patients had died of the disease, and 23 patients were alive with disease. Figure 1 shows the PFS and OS curves for the 216 patients with OC, according to their GPS at pretreatment. The Kaplan–Meier curves showed that the PFS and OS for patients with high pretreatment GPS (GPS 2) were shorter than for patients with lower GPS (GPS 0 + 1) for early‐, advanced‐ and all‐stage OC (OS: *P *< 0.001 for early‐ and all‐stage, *P *= 0.015 for advanced‐stage; PFS: *P *< 0.001 for all three stage groups; Fig. 1).

The correlations between clinical factors and PFS or OS were assessed in univariate and multivariate analyses on early‐, advanced‐stage (Table 3) and all‐stage (Table 4). In univariate analysis of PFS, histology (*P *= 0.033) and GPS (*P *< 0.001) were significantly associated with PFS in early‐stage OC; whereas CA125 (*P *= 0.002), neoadjuvant chemotherapy (*P *= 0.022), lymphadenectomy (*P *= 0.024), and GPS (*P *= 0.001) were significantly associated with PFS in advanced‐stage OC. Furthermore, CA125, lymph node metastasis, no residual tumor (R0), ascites, neoadjuvant chemotherapy, histology, stage, lymphadenectomy, and GPS (*P *< 0.001 for all) were significantly associated with PFS in the all‐stage OC grouping. Univariate analysis showed GPS to be significantly associated with OS in early‐stage OC (*P *< 0.001); whereas no residual tumor (R0) (*P *= 0.005), lymphadenectomy (*P *= 0.047) and GPS (*P *= 0.015) were significantly associated with OS in advanced‐stage OC. We also found CA125, lymph node metastasis, no residual tumor (R0), ascites, neoadjuvant chemotherapy, histology, stage, lymphadenectomy, GPS were significantly associated with OS in all‐stage OC (*P *< 0.001 for all).

**Table 3:** Prognostic factors for progression‐free survival and overall survival with early‐ and advanced‐stage of ovarian cancer selected by Cox's univariate and multivariate analysis

	Univariate analysis				Multivariate analysis	
	Haza ratio	95% CI	*P*‐value	Hazard ratio	95% CI	*P*‐value
Progression‐free survival						
Early Stage (Stage I–II)						
CA125 (≥307.6 U/mL)	2.617	0.701–9.767	0.152			
Histology	4.199	1.121–15.722	0.033^a^	5.072	1.272–20.223	0.021^a^
Lymphadenectomy	1.238	0.310–4.951	0.763			
GPS	16.874	4.173–68.233	<0.001^a^	20.501	4.709–89.240	<0.001^a^
Advance Stage (Stage III–IV)						
CA125 (≥307.6 U/mL)	2.55	1.401–4.639	0.002^a^	2.107	1.140–3.893	0.017^a^
Lymph node metastasis	1.386	0.878–2.188	0.161			
No residual tumor (R0)	1.635	0.919–2.911	0.095			
Ascites	1.561	0.991–2.459	0.055			
Neoadjuvant chemotherapy	2.116	1.114–4.020	0.022^a^	1.95	1.016–3.739	0.045^a^
Histology	1.294	0.563–2.973	0.544			
Lymphadenectomy	1.659	1.070–2.570	0.024^a^	1.691	1.088–2.630	0.020^a^
GPS	2.247	1.400–3.608	0.001^a^	2.015	1.238–3.279	0.005^a^
Overall Survival						
Early Stage (Stage I–II)						
CA125 (≥307.6 U/mL)	3.019	0.609–14.965	0.176			
Histology	2.326	0.426–12.704	0.33			
Lymphadenectomy	1.281	0.234–7.010	0.775			
GPS	28.21	5.586–142.468	<0.001^a^			
Advance Stage (Stage III–IV)						
CA125 (≥307.6 U/mL)	1.839	0.935–3.620	0.078			
Lymph node metastasis	1.642	0.948–2.843	0.077			
No residual tumor (R0)	2.419	1.303–4.429	0.005^a^	2.266	1.191–4.310	0.013^a^
Ascites	1.37	0.807–2.325	0.244			
Neoadjuvant chemotherapy	2.021	0.951–4.295	0.067			
Histology	0.921	0.333–2.550	0.874			
Lymphadenectomy	1.663	1.006–2.749	0.047^a^	1.626	0.958–2.759	0.071
GPS	1.996	1.147–3.475	0.015^a^	2.331	1.317–4.127	0.004^a^

GPS, glasgow prognostic score.

a *P* < 0.05.

**Table 4:** Prognostic factors for progression‐free survival and overall survival with all stage of ovarian cancer selected by Cox's univariate and multivariate analysis

	Univariate analysis				Multivariate analysis	
	Hazard ratio	95% CI	*P*‐value	Hazard ratio	95% CI	*P*‐value
Progression‐free survival						
CA125 (≥307.6 U/mL)	5.882	3.505–9.874	<0.001^a^	1.921	1.073–3.439	0.028^a^
Lymph node metastasis	4.812	3.154–7.340	<0.001^a^	1.654	1.022–2.677	0.040^a^
No residual tumor (R0)	3.721	2.096–6.607	<0.001^a^	1.15	0.620–2.134	0.658
Ascites	3.947	2.521–6.178	<0.001^a^	1.291	0.781–2.134	0.319
Neoadjuvant chemotherapy	8.623	5.202–14.295	<0.001^a^	2.041	1.046–3.982	0.036^a^
Histology	8.643	4.591–16.271	<0.001^a^	1.977	0.818–4.777	0.13
Stage	14.074	7.043–28.124	<0.001^a^	2.064	0.634–6.723	0.229
Lymphadenectomy	2.053	1.362–3.094	<0.001^a^	1.944	1.254–3.014	0.003^a^
GPS	4.717	3.003–7.408	<0.001^a^	2.158	1.325–3.514	0.002^a^
Overall Survival						
CA125 (≥307.6 U/mL)	4.526	2.473–8.284	<0.001^a^	1.366	0.683–2.735	0.374
Lymph node metastasis	4.585	2.785–7.549	<0.001^a^	1.3548	0.762–2.421	0.3
No residual tumor (R0)	4.83	2.624–8.891	<0.001^a^	2.296	1.147–4.597	0.019^a^
Ascites	2.966	1.766–4.979	<0.001^a^	0.929	0.501–1.720	0.814
Neoadjuvant chemotherapy	6.685	3.704–12.065	<0.001^a^	1.838	0.832–4.059	0.132
Histology	6.431	3.074–13.454	<0.001^a^	1.582	0.559–4.476	0.388
Stage	11.451	4.948–26.501	<0.001^a^	2.417	0.590–9.907	0.22
Lymphadenectomy	2.113	1.315–3.396	<0.001^a^	1.6	0.967–2.648	0.067
GPS	4.051	2.405–6.822	<0.001^a^	2.685	1.482–4.865	0.001^a^

GPS, glasgow prognostic score.

a *P* < 0.05.

In multivariate analysis, histology (*P *= 0.021) and GPS (*P *< 0.001) were significantly associated with PFS in early‐stage OC. CA125 (*P *= 0.017), neoadjuvant chemotherapy (*P *= 0.045), lymphadenectomy (*P *= 0.020) and GPS (*P *= 0.005) were significantly associated with PFS in advanced‐stage OC. Furthermore, CA125 (*P *= 0.028), lymph node metastasis (*P *= 0.040), neoadjuvant chemotherapy (*P *= 0.036), lymphadenectomy (*P *= 0.003), and GPS (*P *= 0.002) were significantly associated with PFS in all‐stage OC. Multivariate analysis of OS results showed no residual tumor (R0) (*P *= 0.013) and GPS (*P *= 0.004) are significantly associated with OS in advanced‐stage OC; and no residual tumor (R0) (*P *= 0.019) and GPS (*P *= 0.001) were significantly associated with OS in advanced‐stage of OCs. In particular, the multivariate analysis showed GPS to be independent predictors of recurrence and survival in patients with OC.

## Discussion

The known prognostic factors for OC include residual tumor and chemotherapy response 4, 5. Inflammatory markers such as GPS are important prognostic factors in various cancers. This is the first study to evaluate whether high pretreatment GPS predicts poor prognosis for patients with epithelial OC, including those with early‐stage disease.

Increased CRP may be due to the production of inflammation‐related cytokines such as vascular endothelial growth factor and interleukin (IL)‐6 19, 20. Hefler and colleagues reported that OC patients with low CRP (≤1.0 mg/dL) had significantly better prognoses than those with elevated CRP (>1.0 mg/dL) 21. Hypoalbuminemia is often observed in patients with advanced cancer, and is usually regarded as a marker for malnutrition and cachexia. The low albumin concentration is accordance with proinflammatory cytokines such as IL‐1, IL‐6, and Tumor Necrosis Factor, which modulate albumin production 22, 23. The mechanisms that potentially underlie the relationship between hypoalbuminemia and poor prognosis are similar to those described above for CRP. Reportedly, albumin participates in systemic inflammatory responses and is a prognostic factor for shorter long term survival in patients with various cancer types 24. Warwick and colleagues reported that OC patients with low albumin (<3.5 g/dL) had significantly poorer prognoses than those with an elevated albumin (≥3.5 g/dL) 25. The combination of CRP and albumin in the GPS may reflect both the presence of a systemic inflammatory response and progressive nutritional decline in cancer patients. Sharma and colleagues reported that high GPS is a predictor of poor prognosis for patients with advanced‐stage epithelial OC 16. We investigated whether pretreatment clinical characteristics were correlated with GPS at any stage of OC, and found that pretreatment GPS was significantly associated with stage, histology, lymph node metastasis, macroscopic tumor free (R0), ascites, neoadjuvant chemotherapy and CA125 at all disease stages.

This study investigated whether inflammatory markers of the GPS predicted PFS and OS for patients with all cases of epithelial OC. The median PFS and OS of the high‐GPS patients were significantly shorter than for the low‐GPS group, for early‐, advanced‐ and all‐stage OC. Moreover, multivariate analysis of our study population showed that high pretreatment GPS independently predicted shorter PFS in early‐, advanced‐ and all‐stage OC; and shorter OS in advanced‐ and all‐stage OC. Therefore, determination of GPS at pretreatment may be useful in projecting prognosis of patients at all stages of epithelial OC, including early‐stages disease.

We acknowledge that our study has some limitations. The number of patients was relatively small, and the duration of follow‐up was relatively short. Further prospective studies with more patients and longer follow‐up periods would provide more definitive data to clarify the significance of our findings.

In conclusion, this report shows that high GPS can serve as an indicator of poor prognosis in patients with all stages and histologies of epithelial OC, including early‐stage disease.

## Conflict of Interest

None delared.
